# Prediction of fluid responsiveness by dynamic preload parameters in children undergoing thoracoscopic surgery with one-lung ventilation – A prospective observational study

**DOI:** 10.1097/MD.0000000000031795

**Published:** 2022-12-09

**Authors:** Siyuan Xie, Ding Han, Gang Chen, Shoudong Pan

**Affiliations:** a Department of Anesthesia, Capital Institute of Pediatrics Affiliated Children’s Hospital, Beijing, China.

**Keywords:** children, fluid responsiveness, one-lung ventilation, pulse pressure variation, stroke volume variation

## Abstract

Optimal perioperative fluid management is essential for reducing complications in children undergoing thoracoscopic surgery. The study aimed to assess the performance of 2 dynamic preload parameters – pulse pressure variation (PPV) and stroke volume variation (SVV)- either used alone or combined into a multivariable regression model for predicting fluid responsiveness in children undergoing video-assisted thoracoscopic surgery with one-lung ventilation. Children aged 1 to 6 years old undergoing video-assisted pulmonary segmentectomy or lobectomy were enrolled. Volume loading with 5 mL/kg of hydroxyethyl starch was administered over 15 minutes after establishment of artificial pneumothorax. PPV, SVV, cardiac index, cardiac cycle efficiency, and the difference between systolic blood pressure and dicrotic pressure were recorded using the pressure recording analytical method before and after volume loading. Patients with an elevation in cardiac index greater than 10% were defined as responders, and the remaining patients were nonresponders. Of 40 children, 36 were included in the final analysis, containing 13 responders and 23 nonresponders. SVV had an accuracy of 74% (95% confidence interval, 55–93%) for predicting fluid responsiveness, and a best cutoff of 22% showed a sensitivity of 62% and a specificity of 96%. PPV was incapable of discriminating responders from nonresponders. The multivariate regression model did not perform better than SVV alone. We found PPV failed to predict fluid responsiveness, while SVV predicted fluid responsiveness reasonably in the present context. There was no enhancement in predictivity accuracy with multivariable regression models. The accuracy of these approaches was limited, and more discriminative methods need to be found.

## 1. Introduction

Video-assisted thoracic surgery is increasingly performed in children due to its many advantages over open surgery, including less invasiveness and faster postoperative recovery. Optimal perioperative fluid management plays an important role in enhancing recovery after thoracic surgery. Either hypovolemia or hypervolemia may result in an increased risk of complications and delayed discharge.^[1]^ Goal-directed fluid therapy has been recommended to optimize fluid management.^[2]^

Fluid responsiveness (FR) is commonly employed to determine fluid status in clinical settings by assessing cardiac output elevation in response to volume loading.^[3]^ Various hemodynamic parameters have been assessed as predictors of FR. Dynamic preload parameter based on heart-lung interaction during mechanical ventilation, such as stroke volume variation (SVV) and pulse pressure variation (PPV), has been increasingly used for FR prediction in various clinical settings. The performance of FR prediction by dynamic preload parameter varies according to different clinical settings in children, seeming to be more reliable in children undergoing cardiac surgery.^[4–6]^ The accuracy of dynamic preload parameter for predicting FR has been validated in adults undergoing one-lung ventilation.^[7–9]^ However, there are no literature reports to investigate the performance of dynamic preload parameter for predicting FR in children undergoing video-assisted thoracic surgery with one-lung ventilation. An investigation on this topic could provide useful advice on the value of dynamic preload parameters to the anesthesiologists, ICU physicians, and anesthesia or ICU nurses. Critical care team including anesthesia and ICU nurses could use dynamic preload parameters to monitor and maintain fluid balance, improving the safety and efficacy of fluid therapy.

Various pulse contour methods could provide dynamic preload parameter, including the MostCare device (core technique pressure recording analytical method). Based on mathematical analysis of the arterial waveform, MostCare provides dynamic preload parameter and other hemodynamic parameters such as cardiac output, dicrotic pressure, and cardiac cycle efficiency.^[10]^ Dicrotic pressure corresponds to the transient pressure at dicrotic notch in the arterial waveform. Cardiac cycle efficiency is a parameter representing cardiovascular performance from an energic view. In a recent report, the authors investigated the accuracy of PPV derived from MostCare for predicting FR and found an enhanced accuracy when a multivariate logistic regression model containing dicrotic pressure and cardiac cycle efficiency was employed.^[10,11]^ The outstanding advantage of MostCare is the provision of real-time and continuous assessment of hemodynamic variables, allowing tracking the cardiac output change after a fluid challenge.^[10]^

In this study, we aimed to investigate whether dynamic preload parameter alone or in combination with other hemodynamic parameters could predict FR in children undergoing video-assisted thoracic surgery.

## 2. Materials and methods

### 2.1. Study population

The Institutional Review Board of Capital Institute of Pediatrics approved this study. Written informed consent was obtained from the parents of the participants. From January to December 2019, children undergoing video-assisted pulmonary segmentectomy or lobectomy were considered to be eligible for inclusion in the study. The inclusion criteria were: age between 1 and 6 years old, American Society of Anesthesiologists physical status I-III category, insertion of arterial and central venous catheters, hypotension (a decrease of systolic blood pressure more than 20 mm Hg or 20% from that after anesthesia induction; or systolic blood pressure below age-adjusted values [2 × age + 70 mm Hg]). Exclusion criteria were: body mass index greater than 25 kg/m^2^, significant heart malformations, tachycardia (more than 160 beats/min), cardiac dysrhythmias, arterial waveform artifacts (over-and under-damping, misidentification of dicrotic notch), need for inotropic and vasoactive agents, declining to participate.

### 2.2. Direct systemic hemodynamic monitoring using mostcare

The design of MostCare (Vygon, Vytech) and its algorithm to calculate PPV have been described elsewhere.^[12]^ The device was connected to a standard arterial line through a dual output pressure module in a routine monitor system (Philips). It provided averaged beat-to-beat calculated hemodynamic variables from the last 30 seconds and displayed them continuously. Each parameter was recorded throughout the study period every 30 seconds and was downloaded by dedicated software to Excel sheets for offline analysis. Misidentification of dicrotic notch was checked by visual inspection. A fast flush test was employed to check arterial waveform artifacts.^[13]^ The final data for analysis were the average of 4 readings in 2 minutes during predetermined time points (pre- and post-volume loading).

### 2.3. Study procedure

After admission to the operating room, patients were monitored with 3-lead electrocardiography, digital pulse oximetry, and noninvasive blood pressure. Following induction of anesthesia, patients were intubated with a single lumen endotracheal tube, and bilateral lung ventilation was initiated with a tidal volume of 8 mL/kg. All patients were instrumented with a radial arterial catheter to allow routine arterial pressure monitoring and advanced hemodynamic monitoring with MostCare device. A 5.5 Fr central venous catheter (Arrow International, Inc) was placed into the right internal jugular vein. Anesthesia was maintained with propofol 6 to 10 mg/kg/h, remifentanil 0.2 to 0.3 μg/kg/min, and expired sevoflurane 1% to 2%, targeting a BIS value between 50 and 60. After lateral decubitus positioning, the lungs were isolated by inserting the single-lumen endotracheal tube into one lung under fiberoptic bronchoscopic guidance. Auscultation of lungs confirmed the proper location of endotracheal tube. The pressure-controlled ventilation parameters were: tidal volume 6 mL/kg, positive end expiratory pressure (PEEP) 5 cm H_2_O, fraction of inspired oxygen of 0.6 to 1.0, and the inspiratory to expiratory ratio at 1:1.5. The respiratory rate was adjusted to maintain end-tidal partial pressure of CO_2_ between 40 and 50 mm Hg. The pressure transducers were positioned at heart level and zero balanced after the patients were positioned in the lateral decubitus position. Artificial CO_2_ pneumothorax was established with an insufflation pressure of 4 mm Hg. Hemodynamic variables were allowed to reach steady state for 2 minutes. For eligible participants, volume loading with a 5 mL/kg of hydroxyethyl starch (Voluven, HES 130 kDa/0.4) was administered over 15 minutes.

### 2.4. Outcome variables and study time points

Heart rate, systolic blood pressure (P_sys_), dicrotic blood pressure (P_dic_), PPV, SVV, cardiac cycle efficiency, cardiac index, dp/dt_max_ (the maximal slope of systolic upstroke), systemic vascular resistance index, and stroke volume index were recorded immediately pre- and post-volume loading. Patients were defined as responders to volume loading if there was a greater than 10% increase in cardiac index, and the remaining patients were defined as nonresponders.

### 2.5. Statistical analysis

Statistics were computed using SPSS 20.0 package (IBM, Inc). Receiver operating characteristic curves and dot plot graphics were established using Sigmaplot 12.0 (Systat Software, Inc). The unpaired *t* test for continuous variables and the chi-square test for categorical variables were used to compare data between responders and nonresponders. Paired *t* test was used to assess the difference in hemodynamic parameters before and after volume loading within each group. The receiver operating characteristic analysis was used to assess the accuracy, sensitivity, specificity, and cutoff value of dynamic preload parameter for predicting FR, with the area under the curve representing the accuracy. The cutoff value was defined as the value corresponding to the maximum sum of sensitivity and specificity.

The multivariable logistic regression model was planned to be built in two steps. First, predictors were selected based on significance (*P* < .1) on univariate analysis. Second, the significant predictors were put into the multivariable logistic model using the forward likelihood ratio method.

A *P* value less than .05 was considered statistically significant unless otherwise specified.

## 3. Results

**Figure 1.**
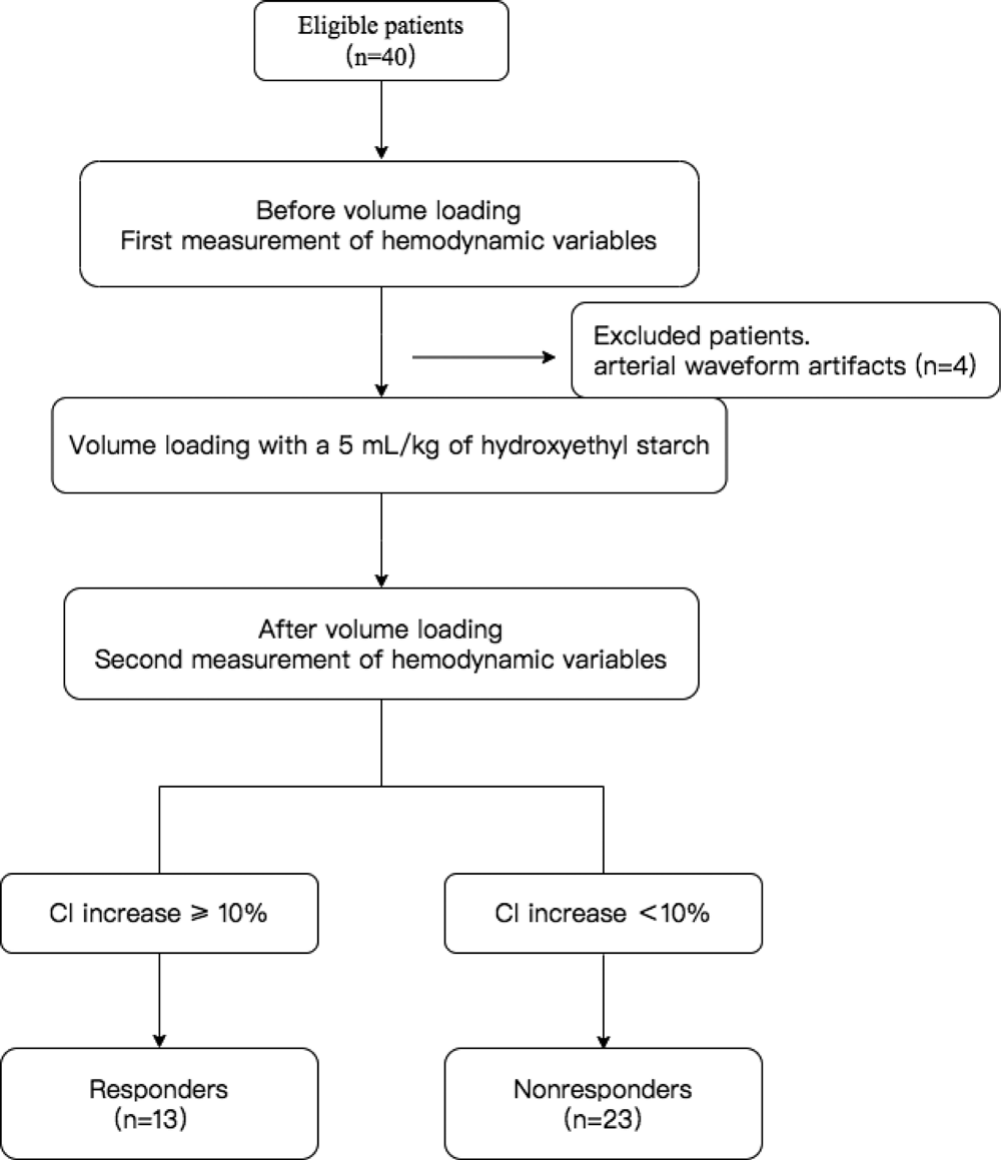
Flowchart. CI = cardiac index.

Of 40 children, 36 were included in the final analysis, excluding 4 with arterial waveform artifacts. Flowchart of the study was shown in Figure 1. All patients went through surgery uneventfully. No inotropic and vasoactive agents were used during the study period. General information of the 36 patients was presented in Table 1. There were no significant differences in the demographic and clinical parameters between responders and nonresponders (*P* > .05).

**Table 1:** Patient characteristics.

Variables	Responders n = 13	Nonresponders n = 23	*P* value
Age (yr)	2.7 ± 1.3	2.4 ± 1.4	.649
Body weight (kg)	11.5 ± 3.8	11.8 ± 3.3	.878
Sex (F/M)	8/5	10/13	.298
ASA I/II/III, n (%)	3/6/4	2/14/7	.471
Left/right lateral decubitus	4/9	9/14	.727

ASA = American Society of Anesthesiologists.

### 3.1. Predictivity of FR

**Figure 2.**
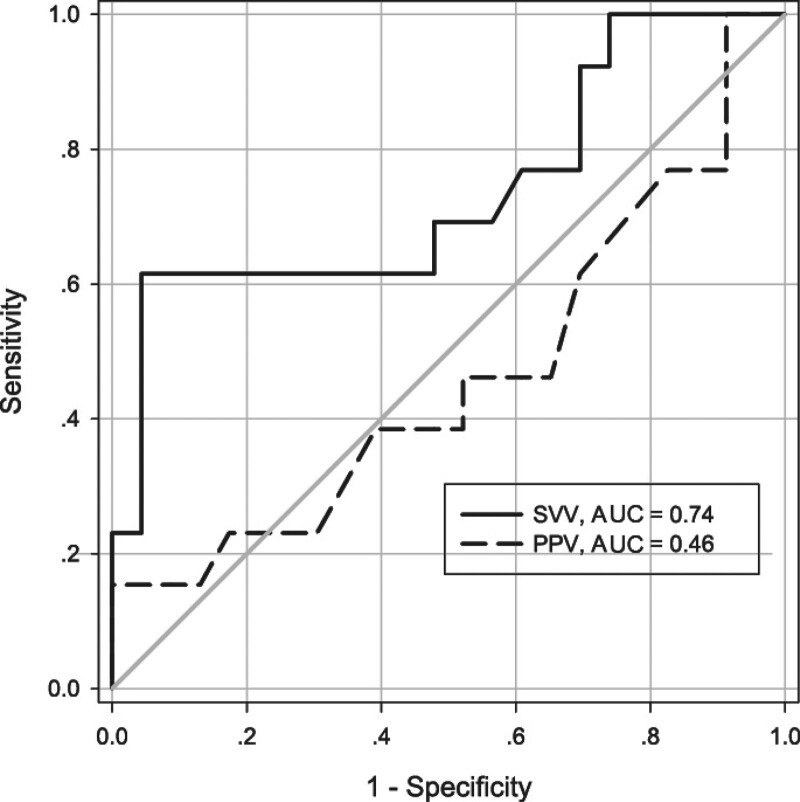
Receiver operating characteristic curves showing the ability of SVV and PPV to predict fluid responsiveness. AUC = area under the curve, PPV = pulse pressure variation, SVV = stroke volume variation.

**Figure 3.**
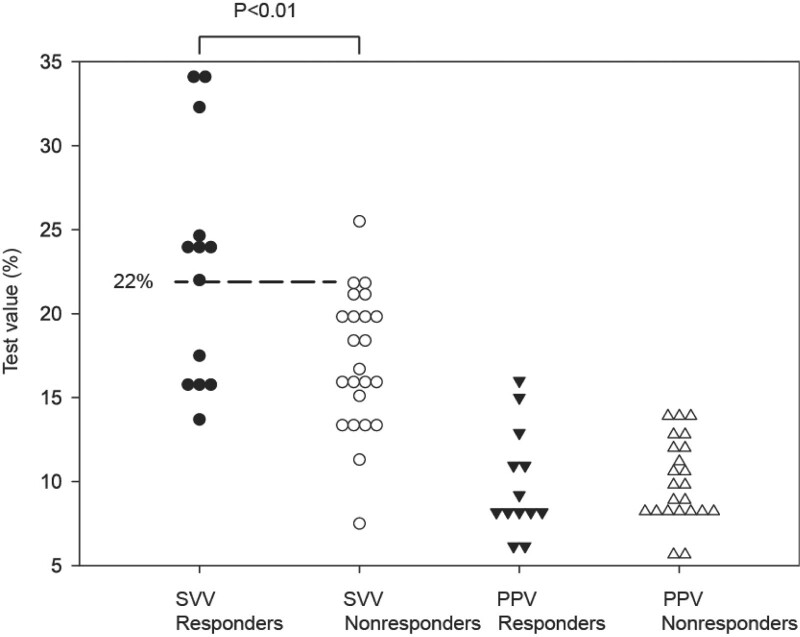
Individual values of SVV and PPV before volume loading in responders and nonresponders. PPV = pulse pressure variation, SVV = stroke volume variation.

According to FR (magnitude of cardiac index elevation) after the volume loading, 13 patients were recognized as responders (cardiac index elevation greater than 10%) and 23 as nonresponders (cardiac index elevation less than or equal to 10%). SVV accurately predicted FR in 73% of subjects, with a cutoff value of 22%, a sensitivity of 61.5%, and a specificity of 95.7%. However, PPV showed poor performance to predict FR, with an accuracy of only 46% (Fig. 2). The individual values of PPV and SVV before volume loading in responders and nonresponders were shown in Fig. 3.

According to the univariate analysis, only SVV met the criteria to be put into the multivariable logistic regression model and therefore the model was not built. Irrespective of the univariate analysis, after entering all predictors into the multivariable logistic regression, only SVV was retained.

### 3.2. Comparison of hemodynamic parameters

Table 2 shows the comparative results of hemodynamic parameters. Except for SVV, all baseline (before volume loading) hemodynamic parameters did not differ significantly between the responders and nonresponders (*P* > .05). For comparisons after volume loading, responders had higher cardiac index, SVV, and stroke volume index, and lower heart rate than nonresponders (*P* < .05 for all).

**Table 2:** Hemodynamic parameters recorded before and after volume loading in responders and nonresponders.

Variables		Before	After	*P*^b^ value
CCE (unit)	Responders	−0.12 ± 0.32	0.00 ± 0.20	.029
	Nonresponders	−0.22 ± 0.35	−0.18 ± 0.33	.181
	*P*^a^ value	.410	.093	
CI (L/min/m^2^)	Responders	3.0 ± 0.6	3.7 ± 0.7	<.001
	Nonresponders	3.1 ± 0.4	3.0 ± 0.4	.091
	*P*^a^ value	.717	<.001	
dp/dt_max_ (mm Hg/ms)	Responders	0.95 ± 0.22	1.03 ± 0.22	.016
	Nonresponders	0.90 ± 0.19	0.96 ± 0.20	.044
	*P*^a^ value	.486	.299	
P_sys_ − P_dic_ (mm Hg)	Responders	27 ± 7	31 ± 8	<.001
	Nonresponders	25 ± 6	26 ± 8	.341
	*P*^a^ value	.416	.111	
P_sys_ (mm Hg)	Responders	85 ± 9	94 ± 11	<.001
	Nonresponders	86 ± 7	90 ± 8	.013
	*P*^a^ value	.774	.263	
PPV (%)	Responders	10 ± 3	7 ± 1	.010
	Nonresponders	10 ± 2	7 ± 2	<.001
	*P*^a^ value	.895	.275	
SVV (%)	Responders	23 ± 7	18 ± 9	.043
	Nonresponders	17 ± 4	13 ± 5	<.001
	*P*^a^ value	.004	.026	
SVRI (dynes/cm^−5^ m^2^)	Responders	1514 ± 242	1456 ± 349	.512
	Nonresponders	1463 ± 246	1515 ± 238	.086
	*P*^a^ value	.552	.553	
Heart rate (beats/min)	Responders	120 ± 18	115 ± 15	.075
	Nonresponders	126 ± 17	129 ± 19	.025
	*P*^a^ value	.375	.041	
SVI (mL/m^2^)	Responders	26 ± 6	32 ± 7	<.001
	Nonresponders	25 ± 8	23 ± 9	.034
	*P*^a^ value	.719	.004	

CCE = cardiac cycle efficiency, CI = cardiac index, dp/dtmax = maximal slope of systolic upstroke, *P*a = compared with nonresponders, *P*b = compared with before volume loading, Pdic = dicrotic pressure, PPV = pulse pressure variation, Psys = systolic blood pressure, SVI = stroke volume index, SVRI = sustemic vascular resistance index, SVV = stroke volume variation.

For intragroup comparison from pre- to post-volume loading, there was an increase in cardiac cycle efficiency, cardiac index, dp/dt_max_, P_sys_ − P_dic_, P_sys_, and stroke volume index (*P* < .05 for all), and a reduction in PPV and SVV (*P* = .010 and .043) in the responders. In the nonresponders, there was an increase in P_sys_, heart rate, and dp/dt_max_ (*P* < .05 for both), and a reduction in PPV, SVV, and stroke volume index (*P* < .05 for all) from baseline (pre-volume loading).

## 4. Discussion

Our study demonstrated that SVV, though with a limited accuracy, but not PPV was predictive of FR in children undergoing video-assisted thoracic surgery with one-lung ventilation. There was no multivariable regression model performing better than SVV alone for FR prediction.

Artificial pneumothorax has significant effects on the cardiovascular system, such as compression of large intrathoracic veins, ventricular compliance reduction, ventricular afterload alternation. The situation is further complicated by one-lung ventilation as it leads to a 20% to 30% shunt through the non-ventilated lung.^[14]^ The principle of dynamic preload parameter generation is based on cyclical loading and unloading of the heart caused by mechanical ventilation. Under one-lung ventilation, the ventilated lung is responsible for the cyclical loading and unloading tasks and allows the principle to work. On this ground, some authors found that dynamic preload parameter was predictive of FR in adults under one-lung ventilation.^[7,9]^

The predictivity of FR by dynamic preload parameter in our results was not remarkable and was only slightly better than, or similar to, that in two similar studies.^[8,14]^ Some of the most common causes for low FR predictivity merit discussion. First, the unsatisfied FR performance of dynamic preload parameter could be ventricular compliance reduction. Artificial pneumothorax led to intrathoracic pressure elevation, constrained the heart relaxation, and limited ventricular ability to increase cardiac output by increasing preload. Second, one apparent reason for the inaccurate FR prediction of PPV was the low magnitude of baseline PPV values. Tidal volume is an important factor that influences the magnitude of dynamic preload parameter. The magnitude of dynamic preload parameter increases with increasing tidal volume and PEEP.^[15]^ In an adult study, a minimum tidal volume of 8 mL/kg was required to induce a significant dynamic preload parameter that allowed for predicting FR during one-lung ventilation.^[16]^ In contrast, another study in adults found that PPV was only predictive of FR during protective ventilation with tidal volume 6 mL/kg plus PEEP at 5 cm H_2_O, but not conventional ventilation with tidal volume of 10 mL/kg. Nowadays, lung protective ventilation strategy using low tidal volume plus PEEP is favored in children requiring one-lung ventilation because of lower postoperative pulmonary complications.^[17]^ Hence we used a small tidal volume of 6 mL/kg with PEEP at 5 cm H_2_O in the present study. Interestingly, we found that PPV values were significantly lower than SVV values in our study. Pulse pressure variation is a surrogate marker of SVV stemmed from variations in the product of downstream arterial blood flow and vascular resistance. Therefore, PPV could be inherently lower than SVV under a relatively normal arterial tone. This is more likely so in children as they have a high arterial compliance.

To improve FR prediction accuracy, PPV and SVV have been combined into multivariable models, of which cardiac cycle efficiency and P_sys_ − P_dic_ have been introduced as auxiliary predictors in previous studies.^[10,11]^ Cardiac cycle efficiency is a cardiovascular performance variable determined by the ratio between mechanical work performed by the heart and the energy expenditure.^[13]^ Changes of cardiac cycle efficiency could access whether a volume loading or unloading is beneficial or detrimental to cardiovascular performance.^[11]^ We did not see a deterioration of cardiac cycle efficiency following volume loading in the nonresponders, possibly due to a proper amount of fluid avoiding overload was administered. Dicrotic notch is the fusion of the forward waves and the retrograde waves generated at the branches of arterioles, corresponding aortic valve closure. Dicrotic pressure increases increases with volume loading in a preload-dependent heart. An increase in P_sys_ − P_dic_ following volume loading relies on the greater increase in P_sys_, standing for an elevation of stroke volume.^[11]^ This matches our observation in the responders. Our results showed that either cardiac cycle efficiency or P_sys_ − P_dic_ was insufficient to identify responders from nonresponders in the present population, potentially because physiological changes of circulation under one-lung ventilation, or that pediatric patients are very different from adults in cardiovascular properties.

Another noted finding of our study was that a cutoff of 22% for SVV achieved high specificity. Based on this finding, volume replacement should be titrated with extreme caution in patients having SVV below 22% while being susceptible to fluid overload.

The study had several limitations. First, the MostCare device has limitations in itself. An over-or under-damping signal from arterial transducer reduces the accuracy of calculating cardiac output. We used a fast flush test to preclude that artifacts exits. Regardless of the accuracy of absolute values, the MostCare device provides plausible values in tracking cardiac output changes following volume loading in children.^[5,12]^ Second, we used a volume of 5 mL/kg administered over 15 minutes based on previous studies in pediatric cardiac surgery.^[5]^ This volume should be considered as moderate or small in similar pediatric studies.^[6]^ Nowadays, the mini-fluid challenge with a rapid infusion of a small volume of fluids has been suggested to test FR while protecting patients from fluid overload.^[18]^ Third, we defined an elevation of cardiac index greater than 10% as fluid responders. The most commonly used cutoff values are 10% and 15%,^[6]^ and we chose 10% to increase the number of responders. Forth, the sample size was relatively small but was considered adequate for a pilot study. Finally, we cannot deny that some unknown factors, i.e., comorbidities, level of fluid defecit, might have affected the outcomes.

## 5. Conclusions

In conclusion, we evaluated the ability of PPV and SVV for predicting FR in children undergoing video-assisted thoracic surgery with one-lung ventilation, and we found PPV failed to predict FR while SVV predicted FR reasonably. There was no multivariable regression models to enhance the accuracy for predicting FR. The accuracy of these parameters was limited, and more discriminative approaches need to be found.

## Author contributions

**Data curation:** Siyuan Xie, Ding Han, Gang Chen.

**Formal analysis:** Siyuan Xie, Ding Han.

**Funding acquisition:** Shoudong Pan.

**Investigation:** Siyuan Xie.

**Methodology:** Siyuan Xie.

**Project administration:** Shoudong Pan.

**Resources:** Ding Han.

**Supervision:** Shoudong Pan.

**Writing – original draft:** Siyuan Xie.

**Writing – review & editing:** Ding Han, Shoudong Pan.
